# A Fourfold Male-Specific Lifespan Extension via Canonical Insulin/IGF-1 Signaling

**DOI:** 10.64898/2025.12.02.691904

**Published:** 2025-12-05

**Authors:** Mike Russell, Michelle Lin, Alexander Tate Lasher, Steven N Austad, Liou Y Sun

## Abstract

The insulin/IGF-1 signaling (IIS) pathway is an evolutionary conserved regulator of longevity, and its modulation is a hallmark of aging research. The 1993 ground-breaking report of a *daf-2* mutation (e1370) that reduced IIS and doubled *C. elegans* lifespan in hermaphrodite worms paved the way for molecular approaches to modulating aging. However, the impact of that mutation on the male sex has remained largely unstudied. Here we report that the same mutation extends male lifespan by fourfold, to over 110 days. This extreme longevity is coupled with a dramatic extension of healthspan as well. These findings establish sex not as a secondary variable but as a primary determinant of longevity potential, capable of amplifying the output of a core aging pathway to an astonishing degree. This work provides a new approach or dissecting the interplay between sex and aging and suggests that sex-specific interventions may be critical for developing future anti-aging therapeutics.

## Full Text Availability

The license terms selected by the author(s) for this preprint version do not permit archiving in PMC. The full text is available from the preprint server.

